# Salivary parameters and oral health status amongst adolescents in Mexico

**DOI:** 10.1186/s12903-020-01182-8

**Published:** 2020-07-06

**Authors:** A. E. González-Aragón Pineda, A. García Pérez, F. García-Godoy

**Affiliations:** 1 Faculty of Higher Studies (FES), Iztacala, National Autonomous University of Mexico (UNAM), Avenida de los Barrios Número 1, Colonia Los Reyes Ixtacala, C.P. 54090 Tlalnepantla, Estado de México Mexico; 2 Bioscience Research Center, College of Dentistry, University of Tennessee Health Science Center, Memphis, TN USA

**Keywords:** Dental caries, Saliva analysis, Adolescents, Oral health

## Abstract

**Background:**

In the last years an increased interest in the use of salivary parameters in connection with caries experience and caries prediction has been shown. In schoolchildren investigations are known, where the relationship between caries prevalence and salivary parameters has been assessed, but in the adolescent population studies are scarce. The aim of the study was evaluate of the association among salivary parameters, oral health status and caries experience in adolescents in Mexico.

**Methods:**

A cross-sectional study was conducted on 256 (DMFT≥5) and 165 (DMFT< 5) 12-to- 14-year-old adolescents. From all the adolescents, unstimulated mid-morning saliva samples were collected, after which the salivary flow rate was calculated, and the salivary pH and buffer capacity was measured. The caries was evaluated via the application of the DMFT score. Clinical variables such as oral hygiene and dental calculus were examined in the adolescent’s oral cavity. The adolescents provided data on their personal characteristics by completing a questionnaire, while socioeconomic data were collected from their parents. Descriptive, bivariate and logistic regression model analyses were performed.

**Results:**

The prevalence of caries was 61.1% (DMFT≥5) in permanent dentition, with 72.7% of subjects presenting poor oral hygiene. The mean levels of salivary flow rate, pH, and buffer capacity was significantly lower (*p* < 0.05) in adolescents with caries score of DMFT≥5 than in those with caries score of DMFT < 5. Salivary flow and buffer capacity were higher in boys than in girls. The logistic regression model applied showed that adolescents with a salivary flow rate < 1 ml per min were more likely to present caries [OR = 1.58 (CI95% 1.04–2.40); *p* = 0.033] than adolescents with a flow rate ≥ 1 ml per-min, and that, for each unit of increased pH, the probability of presenting caries reduced by 76% [OR = 0.24 (CI95% 0.10–0.55); *p* = 0.001].

**Conclusion:**

Significant association was found in salivary flow rate, pH and buffer capacity in adolescents with caries (DMFT≥5). In addition to differences of these parameters by sex, the results suggest saliva parameters may act as indicators of caries in adolescents.

## Background

Dental caries is the commonest disease worldwide affecting a large proportion of the population round the globe [1]. The dental tissues demineralization is due to the acidic by-products resulting from the bacterial fermentation of sugar [2]. Some of these organic acidic bacterial products are the acetic, lactic and propionic acids, which have the ability to dissolve hydroxyapatite [3]. The most relevant acid-producing bacterial species are: *Streptococcus mutans*, *Streptococcus Sobrinus*, *Bifidobacterium dentium*, *Bifidobacterium spp*, *Scardovia wiggsiae*, and *lactobacilli* [4]. A complex mixture of fluids produced by various salivary glands and crevicular fluid [5], saliva promotes the natural beneficial relationship between the resident and host oral microbiota, affecting local pH via its damping properties and thus, contributing to the establishment of stable and diverse microbial communities [6, 7]. Saliva also plays an important role in remineralization, providing calcium, phosphate, and fluoride to dental tissue [8, 9].

Salivary flow is an important factor since it favors the cleaning of bacterial substrates, protects the oral surfaces and helping to control the development of caries. A reduced salivary flow is able of producing a cascade of events where the biofilm accumulates and matures, resulting in the acidification of the microenvironment and the selection of acidogenic bacteria [6, 7]. Perhaps the best clinical indicator of saliva’s protective properties would be salivary flow rate, as other salivary parameters (pH and buffer capacity) depend on it [8].

Factors such as age, sex, and inadequate oral hygiene are elements that change the oral environment [10], with the mechanical control of biofilm via proper toothbrushing twice a day considered to help maintain healthy gums, in addition to preventing the development of mature bacterial communities and increasing the probability that the microbiota remains acidogenic-bacteria free [11]. On the other hand, it has been observed that as age increases, the greater the caries experience in permanent dentition due to various risk factors, data on caries experience in adolescents in Mexico have reported prevalence of 80.0% with a DMFT mean of 4.05 (± 3.47) [12], likewise, a longitudinal study of adolescents in Sweden found an increase in the DMFT mean of 4.5 (± 6.1) after 5 years [13].

Although published studies have found an association between the presence of caries and poor salivary parameters (reduced salivary flow rate, and low pH and/or buffer capacity) [14, 15], other studies have found this association to be inconsistent [16]. While its influence is recognized, more evidence of this association is required, thus motivating the objective of this cross-sectional study, which is to evaluate the association among salivary parameters as salivary flow rate, pH, buffer capacity, oral health status and dental caries experience in adolescents in Mexico. The hypothesis proposed in the present study is that the mean levels of salivary flow rate, pH, and buffer capacity will be lower in adolescents with caries scores of DMFT≥5 than in those with caries scores of DMFT < 5. Moreover, salivary parameters such as salivary flow and buffer capacity will be higher in boys compared to girls.

## Methods

### Ethical approval

This cross-sectional study’s research protocol was reviewed and approved by the Ethics Committee of the Faculty of Higher Studies Iztacala at the National Autonomous University of Mexico (CE/FESI/102017/1209). Before any measurements were taken, written informed consent was obtained from the parents/guardians of all participating subjects.

### Characteristics of the study location

This study adhered to a cross-sectional design. Mexico City is made up of 16 territorial demarcations and the chosen demarcation was Gustavo A. Madero, located to the north of the city and was chosen due to the socioeconomic level of the population. The area selected had a population of 1,185,772 inhabitants (13.2% of the total population of Mexico City), of whom, 41.4% over 15 years of age have been educated up to elementary level and 79.2% have access to health services. The *Consejo Nacional de Población* (CONAPO or National Population Council) indicates a medium level of socioeconomic activity in the study area [17]. For convenience, two schools located in the north of Mexico City were selected. The sample size for the present study was set based on the prevalence of caries in this age cohort, as revealed by previous studies [18]. With a power of 0.80, an alpha of 0.05, and an odds ratio equal to 1.80, the required sample size was 415, with 457 participants invited to participate, assuming a 10% non-response rate. A final sample size of 421 subjects, of the 457 invited to participate, was obtained, with 36 subjects excluded. The inclusion criteria were adolescents aged between 12 and 14 years old; of either gender; with written authorization to participate in the study. The exclusion criteria: physically and medically compromised subjects; subjects who were taking medication; and, subjects who did not attend the saliva sampling.

### Variables and data collection

The variables included in the study were as follows: sex (girls/boys); age (in years); toothbrushing frequency (number of times a day) dichotomized into < 2 times a day and ≥ 2 times a day; use of fluoride toothpaste (yes/no); the Simplified Oral Hygiene Index (OHI-S) dichotomized into poor (OHI-S ≥ 2 score), dental calculus (yes/no), and good hygiene (OHI-S < 2 score); flow rate (milliliters per minute) dichotomized into (< 1 ml/min) and (≥ 1 ml/min); pH of stimulated saliva; buffer capacity (high, medium, and low); stimulated salivary flow, obtained in adherence with standard procedures [19]; and, the subject’s mother’s level of education (Primary or less, secondary, Technical or University).

Dental caries was evaluated in permanent dentition using the World Health Organization (WHO) criteria based on the decayed (D), missing (M), and filled (F) teeth (DMFT) score and grouped into two categories (DMFT< 5 or DMFT≥5) [20]. Adolescents having at least five decayed tooth requiring restoration were taken as caries active subjects according to the WHO criteria.

The clinical oral evaluations were conducted by one dentist using dental mirrors (# 5), WHO dental probes, and artificial light, with the subject’s teeth brushed prior to the evaluation. One examiner participated in a pre-study calibration exercise (theoretical training) with the DMFT score. The measurements to be used by the dentist conducting the oral examination had already been standardized with those of a gold standard evaluator (an expert dentist), obtaining Cohen’s kappa coefficients for intra-rater reliability of 0.84 and 0.89 for dental caries and oral hygiene, respectively.

Information on toothbrushing frequency, use of fluoride toothpaste, and the subject’s mother’s level of education were gathered through a standardized self-reported questionnaire.

### Measurement of saliva properties

All the saliva measurements were made and analyzed in the schools, immediately after taking them. Samples were not stored. Subjects were instructed to neither consume food or drinks nor brush their teeth and/or wash their mouth an hour before the measurements were taken, which took place between 8:00 and 10:00 am to reduce the influence of circadian variations on salivary flow mean. The pH of saliva at rest was determined through a strip because it was measured at the beginning, using a small amount of saliva. On the other hand, the stimulated pH was determined through a potentiometer because this was done in the saliva collected period for 5 min. The flow rate and buffer capacity were also measured in stimulated saliva. The process comprised the following steps [19]:

1. pH of saliva at rest: The student was asked to deposit a drop of unstimulated saliva into a collection vessel, with a pH test strip (Saliva-Check Buffer kit GC America Inc., USA) then placed in contact with the saliva, at rest, for 10 s and then cross-referenced with the manufacturer’s standard. The salivary pH was directly estimated using a pH test strip.
2. Stimulated saliva flow rate: Each student was given a piece of flavorless chewing gum and asked to stimulate the production of saliva for 30 s and then swallow the saliva, which was collected every minute for 5 min. A YS series electronic scale (Ohaus Corporation, Mexico City, Mexico) was used, considering the equivalence of 1 mg = 1 ml, to weigh the saliva collected, with the weight then divided by the number of minutes of collection (ml/min).
3. The pH of the stimulated saliva: A potentiometer with a specialized electrode (Starter ST2100; Ohaus Corporation) was used to measure the pH of the stimulated saliva.
4. Buffer capacity: A drop of saliva was applied over the three fields of a buffer test strip (Saliva-Check Buffer kit, GC America Inc., USA) for an expected 2 min reaction, from which the buffer capacity was determined using the reference values provided by the manufacturer (high, medium or low).

### Statistical analysis

First, a descriptive analysis of the population was performed using measures of central tendency and dispersion for the quantitative variables, and frequency and percentage values for the qualitative variables. Bivariate analysis was then performed to establish the relationship between the independent variables in the adolescent subjects with DMFT ≥5 and DMFT < 5, using the Pearson Chi-squared test for categorical variables and the Wilcoxon rank-sum test and Kruskal-Wallis test for numerical variables. The association between salivary parameters (salivary flow, pH, and buffer capacity) and dental caries (DMFT ≥5) was evaluated by means of multiple logistic regression models adjusted for confounders (oral hygiene, toothbrushing frequency and mother’s level of education), with the Odds Ratio (OR) calculated to a 95% Confidence Interval (95% CI). Model diagnostic tests were conducted using the Hosmer-Lemeshow goodness of fit test and the statistical analysis of extreme values, while, finally, possible interactions were analyzed, with values of *p* ≤ 0.05 considered statistically significant, using the Stata 15 program (Stata Corp, College Station, TX, USA).

## Results

Of the 421 participants in the present study, the mean age was 12.3 (±0.46) years, while the percentage of girls and boys examined was 53.0 and 47.0%, respectively. No significant difference was found for mean age by sex (*p* = 0.055). Our findings revealed that 33.7% of the students brushed their teeth twice or more times per day and 66.3% once per day or less and 95.8% using toothpaste, while, according to the OHI-S evaluation, 72.7% of subjects had poor oral hygiene.

The prevalence of caries (DMFT≥5) in permanent dentition was 61.1%, while the mean DMFT score was 5.96 (±3.98), with statistically significant differences observed based on gender (boys 5.1 ± 3.6, and girls 6.6 ± 4.1, *p* < 0.05), the largest component of the DMFT score was decayed teeth with 76.6%, followed by filled teeth with 23.4%. No permanent tooth was recorded as lost. The prevalence of caries was higher in adolescents with poor oral hygiene than adolescents with good oral hygiene (68.0% vs 32.0%, *p* = 0.007). The values for sex, age, toothbrushing frequency, oral hygiene, dental calculus, and the subject’s mother’s level of education are summarized in Table 1.

**Table 1:** Characteristic oral health in adolescents from 12 to 14 years from Mexico

	**DMFT < 5*n*** **= 165**	**DMFT ≥ 5*n*** **= 256**	**Value p**
Age	12.3 (±0.53)	12.4 (±0.55)	0.055^a^
Sex			
Boys	90 (54.6)	108 (42.2)	0.013^b^
Girls	75 (45.4)	148 (57.8)	
Toothbrushing frequency			
< 2 times a day	119 (72.1)	160 (62.5)	0.042^b^
≥ 2 times a day	46 (27.9)	96 (37.5)	
Oral hygiene (OHI-S)			
Good hygiene	33 (20.0)	82 (32.0)	0.007^b^
Poor hygiene	132 (80.0)	174 (68.0)	
Dental Calculus			
No	111 (67.3)	186 (72.7)	0.237^b^
Yes	54 (32.7)	70 (27.3)	
Mother’s level of education			
Primary or less	35 (21.2)	60 (23.4)	0.027^b^
Secondary	53 (32.1)	109 (42.6)	
Technical or University	77 (46.7)	87 (34.0)	

^a^Wilcoxon rank-sum (Mann-Whitney) test ^b^Chi-squared test

Table 2 shows the data for salivary parameters associated with DMFT score. The mean for the salivary pH stimulated was lower in the DMFT ≥5 group than in the DMFT < 5 group (7.2 vs. 7.4; *p* = 0.001, respectively). The prevalence of caries (DMFT≥5) was higher in the decreased salivary flow group (< 1 ml per-min) than in the normal salivary flow group (59.8% vs. 40.2%, respectively; *p* = 0.004). Regarding the buffer capacity, the prevalence of caries (DMFT ≥5) was higher in the low buffer capacity group than in the high buffer capacity group (29.3% vs. 9.0%, respectively; *p* = 0.020). Salivary pH (*r* = − 0.247, *p* = 0.001) and buffer capacity (*r* = − 0.152; *p* = 0.001) findings both showed a significant negative correlation with the DMFT score.

**Table 2:** Distribution of salivary parameters in adolescents from 12 to 14 years with DMFT≥5 vs DMFT< 5 from Mexico

	**DMFT < 5*n*** **= 165**	**DMFT ≥ 5*n*** **= 256**	**p**
	**Mean (SD**^**a**^**)**	**Mean (SD**^**a**^**)**	
Resting pH	7.1 (±0.49)	6.9 (±0.54)	0.001^b^
Salivary pH stimulated	7.4 (±0.25)	7.2 (±0.28)	0.001^b^
Flow rate			
≥ 1 ml per-min	90 (54.6)	103 (40.2)	0.004^c^
< 1 ml per-min	75 (45.4)	153 (59.8)	
Buffer capacity			
High	28 (17.0)	23 (9.0)	0.020^c^
Medium	102 (61.8)	158 (61.7)	
Low	35 (21.2)	75 (29.3)	

^a^*SD* Standard Deviation ^b^Wilcoxon rank-sum (Mann-Whitney) test ^c^Chi-squared test

Table 3 shows that salivary flow and buffer capacity were higher in boys than in girls. Table 4 shows the data related to the multivariate logistic regression analysis conducted for caries (DMFT≥5). Subjects with a toothbrushing frequency < 2 times a day were more likely to present caries [OR = 1.68 (CI95% 1.07–2.63); *p* = 0.023] than those with a toothbrushing frequency ≥ 2 times a day, while subjects with a salivary flow rate < 1 ml per-min were found to be more likely to have caries than those with a salivary flow rate ≥ 1 ml per-min [OR = 1.58 (CI95% 1.04–2.40); *p* = 0.033]. Additionally, the variables of sex, oral hygiene, and the subject’s mother’s educational level were positively associated with the presence of caries (DMFT≥5).

**Table 3:** Distribution of salivary parameters in adolescents from 12 to 14 years from Mexico by sex

	**Boys*n*** **= 198**	**Girls*n*** **= 223**	**p**
	**Mean (SD**^**a**^**)**	**Mean (SD**^**a**^**)**	
Resting pH	6.91 (±0.55)	6.99 (±0.50)	0.038^b^
Salivary pH stimulated	7.29 (±0.28)	7.24 (±0.27)	0.048^b^
Flow rate			
≥ 1 ml per-min	101 (51.0)	92 (41.3)	0.045^c^
< 1 ml per-min	97 (49.0)	131 (58.7)	
Buffer capacity			
High	35 (17.8)	16 (7.1)	< 0.001^c^
Medium	128 (65.0)	134 (59.8)	
Low	34 (17.2)	74 (33.1)	

^a^SD Standard Deviation ^b^Wilcoxon rank-sum (Mann-Whitney) test ^c^Chi-squared test

**Table 4:** Adjusted odds ratios from the logistic regression model for the dental caries (DMFT≥5) in adolescents from 12 to 14 years from Mexico

**Variables**	**Odds Ratio Crude (95%CI)**^**a**^	**p**	**Adjust Odds Ratio (95%CI)**^**a**^	**p**
Age ^b^	1.42 (0.98–2.05)	0.062	1.04 (0.69–1.56)	0.831
Sex ^c^	1.65 (1.11–2.44)	0.012	1.57 (1.03–2.38)	0.035
Oral Hygiene (OHI-S) ^d^	1.88 (1.18–2.99)	0.007	1.69 (1.03–2.74)	0.035
Toothbrushing frequency ^e^	1.57 (1.03–2.40)	0.035	1.68 (1.07–2.63)	0.023
Mother’s level of education ^f^	1.70 (1.14–2.53)	0.009	1.59 (1.05–2.43)	0.030
Salivary pH stimulated ^g^	0.18 (0.08–0.39)	0.001	0.24 (0.10–0.55)	0.001
Flow rate ^h^	1.78 (1.20–2.64)	0.004	1.58 (1.04–2.40)	0.033

^a^OR Odds ratio, *CI* Confidence interval. Reference group: Age^b^ = continuous, Sex^c^ = Boys, OHI-S^d^ = Good, Toothbrushing frequency ^e^ ≥ 2 times a day, Mother’s level of education ^f^ = Technical or University, Salivary pH stimulated ^g^ = continuous, Flow rate ^h^ = ≥ 1 ml per-min

Log likelihood = − 259.45519, Hosmer-Lemeshow = 0.4923

## Discussion

The present study found an association between decreased salivary flow rate (OR = 1.58) and a DMFT≥5 score in subjects, in contrast with adolescents with a DMFT < 5, and, moreover, found that, as the subjects’ salivary pH decreased (OR = 0.24), the probability of caries (DMFT≥5) increased. Various studies have found an association between dental caries and different salivary parameters, including salivary flow rate, buffer capacity, and increased levels of *Streptococcus mutans* [14, 21].

Although the literature reveals controversial results for the associations between salivary parameters and caries, most research has reported a high prevalence of caries in subjects with a decreased salivary flow rate, decreased buffer capacity, and early and/or high colonization counts for *Streptococcus mutans* in saliva [22].

Prabhakar A et al. evaluated the relationship between salivary parameters in children both with and without caries, finding reduced salivary flow, pH, and buffer capacity in children with the presence of caries [23]. Moreover, Gábris K et al. reported a correlation between salivary parameters and the presence of dental caries [24]. The evaluation, in specific age groups (in this instance adolescents), of salivary parameters related to caries is of great importance, as most risk factors have specific consequences for different age groups. Adolescent patients need more comprehensive caries-management strategies in order to avoid the appearance of new incipient and cavitated caries lesions, strategies which include good eating habits, fluoride use, and daily toothbrushing with fluoridated toothpaste, in addition to regular visits to the dentist. All such measures aim to reduce the occurrence of dental caries and positively impact adolescents’ quality of life.

The properties of saliva have an essential role in protecting against caries and preventing the progression of carious lesions, given its buffer capacity and the fact that it helps to wash the dental surface, eliminates bacteria and controls demineralization and remineralization in enamel, in addition to other mechanisms that contribute to protecting dental health [25]. Furthermore, an important function of salivary flow is to dissolve bacterial substrate, wherein, if this flow is diminished, it will not be able to dislodge any of the microorganisms adhered to the epithelial tissue of the oral cavity. Moreover, reduced salivary flow will decrease the protective components contained in saliva, which will result in reduced pH, demineralization, and the advancement of the caries process [14, 26].

In the present study, a decrease in both salivary pH (*p* = 0.001) and buffer capacity (*p* = 0.020) was observed among adolescents with DMFT ≥5, in contrast with adolescents with DMFT < 5. In addition, a significant negative correlation was found between the subject’s DMFT score and their salivary pH and buffer capacity, with this finding similar to results obtained by research conducted in other countries. In a sample of 312 subjects in Saudi Arabia, Farsi reported a relationship between salivary pH and the presence of caries [27]. Similarly, the decreased pH and buffer capacity observed could also be due to intrinsic factors (such as bicarbonate and phosphate levels, and protein buffer systems) [28] or extrinsic factors (such as oral hygiene and food).

Research has reported that the buffer capacity of saliva is altered by metabolic and hormonal changes, as well as the subject’s health in general [26]. The present study found that salivary flow (*p* = 0.045) and buffer capacity (*p* < 0.001) were higher in boys than in girls. Galvão-Moreira et al. investigated the salivary parameters related to caries susceptibility in boys and girls, finding that girls had lower salivary pH values than boys (*p* < 0.05) and noting that gender was a predictor of both salivary flow (R^2^ = 0.05; *p* < 0.05) and pH (R^2^ = 0.16; *p* < 0.001) [29].

The participants in the present study showed a high prevalence of DMFT≥5 score, approximately 61%, which could be a consequence of the subjects’ limited access to health services resulting from their low socioeconomic status. Despite the majority reported used fluoride toothpaste, less than half reported brushing their teeth > 2x/day (33.7%). The fluoride toothpaste has been shown to play an important role in caries prevention, which not only removes plaque, which mostly comprises bacteria that increases the risk of oral disease but can also be used to apply fluoride onto the teeth [30]. The benefits of using fluoride paste to prevent dental caries in children and adolescents have also been demonstrated in a systematic review [31]. In the present study more than 90% mentioned using toothpaste, but approximately less than 50% of children brush >2x/day and 70% have poor hygiene. So, we could say that children have low knowledge about the use of toothpaste, toothbrushing and oral hygiene.

Lack of toothbrushing (OR = 1.68; *p* = 0.023) and poor oral hygiene (OR = 1.69; *p* = 0.035) were associated with the presence of caries in the adolescent subjects of the present study. The accumulation of plaque and the lack of toothbrushing help cariogenic bacteria to pass through and destroy the enamel more quickly, with subjects with poor oral hygiene presenting higher caries rates [32]. Research conducted on a population of adolescents aged between 12 and 15 in Spain reported that 12-year-old individuals who reported occasionally brushing their teeth (OR = 1.83; *p* = 0.028) presented plaque (OR = 2.03; *p* = 0.021) and higher caries values [33], results which were similar to those found in the present study.

Finally, it has been reported that the subject’s mother’s educational level is related to the presence or absence of dental caries [34]. A study undertaken in Italy in a population of four-to-fourteen-year-old children found that the presence of caries was higher in children whose mothers had a lower educational level (OR = 6.1) [34]. The present study found a relationship between caries and a low educational level in the subject’s mother (OR = 1.59).

One of the limitations of this study is the type of convenience sampling used where not all adolescents in the study area were likely to be chosen, another limitation was that salivary buffer capacity was evaluated with strips and not with laboratory tests, which would help to give more precise information. Finally, another limitation in this study is the socio-economic level with the aim of knowing the different economic and social conditions of the population and their distribution with salivary parameters and caries.

## Conclusions

The present study found a significant association in salivary flow rate, pH, and buffer capacity levels in adolescents with caries (DMFT≥5), in addition to observing differences in these parameters by sex. In general, a decreased salivary flow increases the probability of presenting more caries. The results of the present study suggest that the levels of these saliva parameters may be used as indicators of the state of caries in adolescents.

## Data Availability

The datasets used and analyzed during the current study are available from the corresponding author on reasonable request.

## Abbreviations

CONAPO: Consejo Nacional de Población or National Population Council
OHI-S: Simplified Oral Hygiene Index
WHO: World Health Organization
DMFT: Decayed (D), Missing (M), Filled (F), teeth
OR: Odds Ratio
CI: Confidence Interval
